# mSphere of Influence: Celebrating exceptions to the rule of lipid A essentiality

**DOI:** 10.1128/msphere.00633-23

**Published:** 2024-02-29

**Authors:** Katherine R. Hummels

**Affiliations:** 1 Department of Microbiology, University of Georgia, Athens, Georgia, USA; University of Georgia, Athens, Georgia, USA

**Keywords:** cell envelope, outer membrane, lipid A, lipopolysaccharide

## Abstract

Kate Hummels works in the field of bacterial cell envelope biosynthesis and studies the regulation of the metabolic pathways needed to build the Gram-negative cell envelope. In this mSphere of Influence article, she reflects on how the papers “A penicillin-binding protein inhibits selection of colistin-resistant, lipopoligosaccharide-deficient *Acinetobacter baumannii*” by Boll et al. and “*Caulobacter* lipid A is conditionally dispensable in the absence of *fur* and in the presence of anionic sphingolipids” by Zik et al. made an impact on her by studying organisms that deviate from accepted norms to highlight the plethora of unanswered questions in cell envelope biology.

## COMMENTARY

Gram-negative bacteria have a three-layered cell envelope in which an inner and outer membrane sandwich the cell wall. The inner membrane is a glycerophospholipid bilayer, but the canonical Gram-negative outer membrane is an asymmetric bilayer in which the inner leaflet is comprised of glycerophospholipids and the outer leaflet is enriched in the glycolipids lipopolysaccharide (LPS) or lipooligosaccharide (LOS), depending on the species (1). Both LPS and LOS share a conserved hydrophobic anchor called lipid A that is generally thought to be essential for viability (1). There are a subset of Gram-negative species, however, that do not naturally produce lipid A and others in which mutants that do not produce lipid A can be isolated (such as *Acinetobacter baumannii* and *Caulobacter crescentus*) (2–4). It is unclear why some species tolerate loss of lipid A, but these examples underscore the fact that we still have not answered a basic question in the biology of Gram-negative bacteria: if lipid A can be lost, why is it so often essential? In the papers “A penicillin-binding protein inhibits selection of colistin-resistant, lipopoligosaccharide-deficient *Acinetobacter baumannii*” by Boll et al. and “*Caulobacter* lipid A is conditionally dispensable in the absence of *fur* and in the presence of anionic sphingolipids” by Zik et al., the conditional essentiality of lipid A in two distantly related bacteria is leveraged to investigate the factors that are required to support growth in the absence of a canonical Gram-negative outer membrane (3, 5). These studies highlight that, despite decades of research, there are still surprises to be uncovered in the study of a structure as seemingly well conserved as the bacterial cell envelope.

To gain a better understanding of why lipid A is not always required for bacterial viability, Boll and colleagues attempted to select for mutants that abolish lipid A production in a series of 15 *A*. *baumannii* clinical isolates (5). They found that, although many of the isolates supported growth in the absence of lipid A, it was essential in several *A. baumannii* strains. The authors then took advantage of this unique system to ask what was special about the strains that supported the loss of lipid A biosynthesis. Surprisingly, they found that lipid A was only essential if an enzyme involved in synthesis of the cell wall, PBP1A, was highly expressed. The mechanism by which PBP1A impaired growth in the absence of lipid A remained unclear, but the genetic interaction between lipid A and cell wall biosynthesis was unexpected and exciting. Subsequent studies have revealed functional overlap between the outer membrane and cell wall and suggested that fortifications to the *A. baumannii* cell wall can accommodate growth in the absence of lipid A (6, 7). Prior to reading the article by Boll et al., I was under the rather naïve impression that we knew most of what there was to know about lipid A. This work encouraged me to think beyond what we understand from classical model systems and consider what can be learned from the “rule breakers.”

Even though I recognized that there was still much to uncover regarding lipid A biogenesis, I was surprised and excited by the recent report of the conditional essentiality of lipid A in *C. crescentus* by Zik and colleagues (3). In this study, the authors set out to characterize an essential gene of unknown function and wound up identifying a condition in which lipid A is dispensable. In this condition, they found that a sphingolipid is necessary to support growth in the absence of lipid A. Sphingolipids have only been reported in a subset of bacteria and it is still uncertain how they allow for the loss of lipid A, but it is notable that sphingolipids are also produced by *Sphingomonas* spp., a taxon that naturally does not make lipid A (4). *C. crescentus* has been a model system for the study of the bacterial cell cycle and cellular differentiation for decades, but even with that abundance of research, we only now understand that lipid A is conditionally essential in this organism. From this study, Zik and colleagues pushed me to reflect on my own assumptions and recognize that the systems that deviate from the norm may be more common than I previously appreciated.

Despite the similarities in the studies by Boll et al. and Zik et al., they each imparted unique lessons to me which are reflective of the career stage that I was in when they were published. I was a graduate student when the work by Boll and colleagues was published, and, after reading the article, I began to appreciate just how much we did not know about lipid A and sought out other exceptions to the rule. As a result, I decided to spend my postdoctoral career investigating unanswered questions regarding lipid A biosynthesis in *Pseudomonas aeruginosa*, where lipid A production is essential (or, at least, we think it is for now), but under the control of a unique regulatory system (8, 9). In contrast, the work by Zik et. al. was published when I was several years into my postdoc and had been studying lipid A biosynthesis for some time. It served as a reminder that, even if I think that I know the rules of a system, those rules can always change. Although it is still unclear why lipid A is so often essential, I expect that we will continue to uncover more examples of bacterial rule breakers that will help us further resolve this enigma and other important questions regarding cell envelope biosynthesis.
